# A Facile Asymmetric Synthesis of (*S*)-14-Methyl-1-Octadecene, the Sex Pheromone of the Peach Leafminer Moth

**DOI:** 10.3390/molecules18055201

**Published:** 2013-05-07

**Authors:** Tao Zhang, Wei-Li Ma, Tian-Rui Li, Jia Wu, Jun-Run Wang, Zhen-Ting Du

**Affiliations:** 1 College of Sciences, Northwest A&F University Yangling 712100, Shaanxi, China; E-Mails: fuzitong@163.com (T.Z.); maweili@nwsuaf.edu.cn (W.-L.M.); litianrui001@gmail.com (T.-R.L.); jiabaobao1991@gmail.com (J.W.); wangjr07@163.com (J.-R.W.); 2 Key Laboratory of Synthetic Chemistry of Natural Substances, Shanghai Institute of Organic Chemistry, Chinese Academy of Sciences, Shanghai 20032, China

**Keywords:** 14-methyl-1-octadecene, asymmetric synthesis, peach leafminer moth, (*S*)-4-benzyloxazolidin-2-one

## Abstract

An asymmetric synthesis of 14-methyl-1-octadecene, the sex pheromone of the peach leafminer moth has been achieved. The target molecule was synthesized in six linear steps and in 30.3% overall yield from commercially available hexanoyl chloride, (*S*)-4-benzyloxazolidin-2-one and 1,9-nonanediol. The hexanoyl chloride was connected with (*S*)-4-benzyloxazolidin-2-one, and with the induction of the chiral oxazolidinone auxiliary, after chiral methylation, LAH reduction and then tosylation gave the chiral key intermediate **5** in high stereoselectivity. 1,9-Nonanediol, was selectively brominated, THP protected and subjected to Li_2_CuCl_4_-mediated C-C coupling to afford a C_12_ intermediate. The target molecule, (*S*)**-**14-methyl-1-octadecene, was obtained after the two parts were subjected to a second Li_2_CuCl_4_-mediated C-C coupling. Our synthetic approach represents the first time a substrate-control asymmetric synthesis of (*S*)-14-methyl-1-octadecene has been reported.

## 1. Introduction

Nowadays, there is a sharp conflict between intense agricultural production and environmental issues. We all are facing a big challenge caused by the detrimental effects resulting from traditional agricultural production [1], therefore, there is an urgent need to develop more green production modes, such as the use of environmentally benign, low-dosage sex pheromones to control pests [2,3]. This mode plays an important role in so-called Integrated Pest Management (IPM). The peach leafminer moth, *Lyonetia clerkella*, is one of the most destructive pests in peach orchards in East Asia. It causes defoliation when the leaves are infested by only a few larvae of this insect and Chinese orchardists use traditional highly toxic pesticides to control it. We envisaged that an ideal way to control it could be by using its sex pheromone to disrupt its mating process and to trap it at a suitable time.

**Figure 1:**
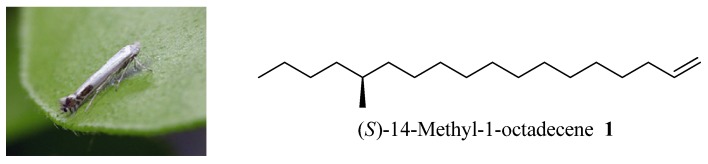
Peach leafminer moth and the chemical structure of its sex pheromone.

The sex pheromone of peach leafminer was first identified as (*S*)-14-methyl-1-octadecene (**1**, Figure 1) by Sugie *et al.* [4] and syntheses of **1** in racemic form have been achieved by two teams [5,6]. Recently, Ando disclosed an informal synthesis of (±)-**1** through a hard-to-get intermediate [7]. The asymmetric syntheses of **1** were also reported by Mori [8] and Kharisov [9], who both used expensive chiral starting materials. However, the existing synthetic pathways have some drawbacks such as requiring too many chemical operations or lack of cost-effectiveness, so the development of a more economical synthesis of **1** in consideration of applying this kind of chemical in the pesticide industry is highly desirable.

Our research group is interested in finding new agrochemicals and lowering the cost of existing lead compounds or pesticides [3,10]. We are interested in an efficient and convenient synthesis of sex pheromones through more economical approaches. Herein, we wish to report a synthesis of (*S*)-14-methyl-1-octadecene (**1**) from inexpensive starting materials by a substrate-induction strategy. 

## 2. Results and Discussion

**Scheme 1:**
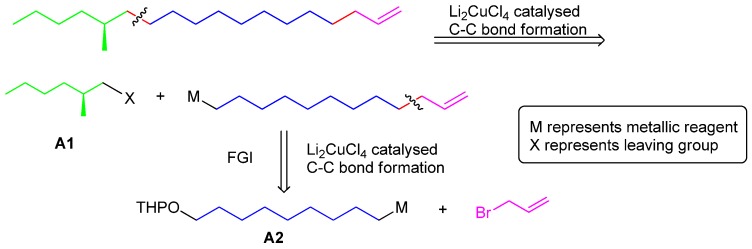
Retrosynthesis of **1**.

The retrosynthesis of **1** is shown in Scheme 1. First, the molecule was disconnected into a chiral C_7_ synthon A1 and a C_12_ moiety. Then the C_12_ moiety in turn could be disconnected into two parts to be joined through a Li_2_CuCl_4_ mediated C-C coupling protocol, a C_9_ subunit **A2** and an allyl bromide which is highly active in S_N_2 reactions and helpful to apply the strategy.

**Scheme 2:**
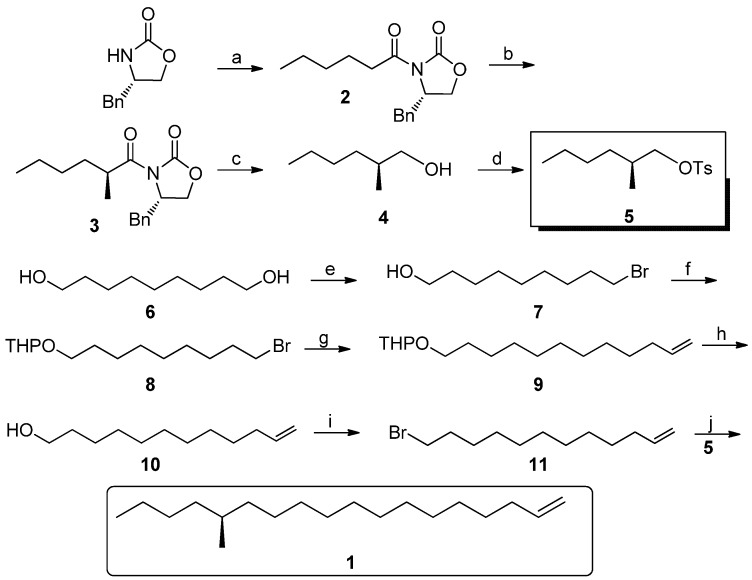
Synthesis of (*S*)-**1**.

As shown in Scheme 2, our synthesis commenced with (*S*)-4-benzyloxazolidin-2-one, which is cheap and can be recycled in industry. After deprotonation, it reacted with hexanoyl chloride to give **2** in 70% yield. Compound **2** was deprotonated at −78 °C in the presence of a strong base such as LDA, then it was stereoselectively methylated to afford **3** [11,12,13]. Because of the steric effect of chiral benzyl group in the oxazolidine ring, the methyl groups was directed to the β-face in d.r. = 1:50 and 78% yield. Then the chiral oxazolidine auxiliary was smoothly removed by LAH reduction at −10 °C. However, in attempting to purify compound **4** in the laboratory, this was found to be very laborious and less effective, so the crude **4** was directly treated with TsCl in pyridine, thus the key intermediate **5** was obtained in 55% yield in two steps. 9-Bromononan-1-ol was prepared in large quantities from nonane-1,9-diol through selective bromination of the α,ω-diol by a known method reported by Kang *et al*. [14]. The hydroxyl group of **7** was protected as its tetrahydropyranyl ether in 92% yield by 3,4-dihydro-2*H*-pyran under acidic conditions. Compound **8** can be converted into a Grignard reagent, and then this was coupled with allyl bromide under Li_2_CuCl_4_ catalysis to form **9** in 66% yield [15,16]. Afterwards, the THP group was removed in methanol in the presence of 4-methyl-benzenesulfonic acid [17,18]. Compound **11** was produced in 90% yield after **10** was subjected to a Corey-Fuji protocol.

*Reagents and conditions*: a) BuLi, −78 °C then hexanoyl chloride, 70%; b) LDA, −78 °C, MeI, 78%, d.r. = 1:50; c) LAH, −10 °C; d) TsCl, Pyr., −10 °C, 15h, 55% for two steps; e) 40% HBr, toluene, I_2_, Dean-Stark trap, 80 °C, 89%; f) TsOH (cat.), DHP, CH_2_Cl_2_ 0 °C - r. t. 92%; g) Mg then Li_2_CuC1_4_, allylic bromide, 0 °C to reflux, 66%; h) CH_3_OH/H_2_O, PPTS (cat.), reflux 3h, 96%; i) PPh_3_, CBr_4_, CH_2_Cl_2_, 90%; j) Mg then Li_2_CuC1_4_, 65%.

With compound **11** in hand, it was converted into the corresponding Grignard reagent and coupled with **5** in the presence of Li_2_CuCl_4_ to afford (*S*)-**1** in 65% yield. Thus, the asymmetric total synthesis of (*S*)-**1** in an overall yield of 30.3% was achieved in six linear steps. Compared with other asymmetric synthesis of the target, our synthesis is apparently more cost-effective, even if the chiral oxazolidinone auxiliary were not recycled.

## 3. Experimental

### General

The ^1^H-NMR and ^13^C-NMR data were recorded in CDCl_3_ solution with Bruker NMR spectrometers (DRX 500, AM 300) if not noted otherwise. The chemical shifts are measured relative to TMS (*δ* = 0) or chloroform (*δ* = 7.26) and the coupling *J* is expressed in Hertz. Mass spectra were recorded on a Thermo Scientific TSQ Quantum Access MAX mass spectrometer (ESI, positive or negative). Standard flash chromatography was employed to purify the crude reaction mixture using 200–300 mesh silica gel (Tsingdao Ocean Company, Tsingdao, China) under a positive nitrogen pressure. Tetrahydrofuran (THF) and diethyl ether were freshly distilled from lithium aluminium hydride under an argon atmosphere. Dichloromethane, hexane and toluene were freshly distilled from calcium hydride under argon.

*(S)-4-Benzyl-3-hexanoyloxazolidin-2-one* (**2**). At −78 °C, to a solution of (*S*)-4-benzyloxazolidin-2-one (5.32 g, 0.03 mol) in anhydrous THF (50 mL) was added BuLi (0.036 mol), then freshly distilled hexanoyl chloride (4.63 g, 0.033 mol) in THF (25 mL) was added after half an hour. The mixture was gradually warmed to 25 °C and maintained for 12 h. After completion, the reaction was quenched by addition of saturated aqueous NH_4_Cl, and the volatiles were evaporated. The residue was extracted with ethyl acetate (100 mL × 3), and the combined organic layers were washed with dilute NaOH and brine, and dried over anhydrous MgSO_4_. Then the extract was evaporated, and purified by column chromatography (15–25% gradient, EtOAc-hexane) to give **2** as a yellowish oil (5.78 g, 70%). ${\left[\alpha \right]}_{D}^{25}$ = +97.6° (c 0.36, MeOH); ^1^H-NMR (500 MHz, CDCl_3_): 0.90 (br s, 3H), 1.34 (br s, 4H), 1.67 (br s, 2H), 2.73–2.77 (m, 1H), 2.84–2.93 (m, 2H), 3.22 (d, *J* = 13.3, 1H), 4.10 (s, 2H), 4.62 (s, 1H), 7.17–7.28 (m,5H); ^13^C-NMR (125 MHz, CDCl_3_): 13.9, 22.4, 23.9, 31.3, 35.4, 37.8, 55.0, 66.1, 127.2, 128.8, 129.4, 135.5, 153.4, 173.2.

*(S)-4-Benzyl-3-((S)-2-methylhexanoyl)oxazolidin-2-one* (**3**). To a cooled (−78 °C) solution of LDA (0.051 mol) was added a solution of compound **2** (11.7 g, 43 mmol) in THF (90 mL). After 2 h, dry MeI (13.3 mL, 0.213 mol) was introduced through a dropping funnel at this temperature. The reaction mixture was allowed to stir at −78 °C for 3 h before being warmed to 25 °C and maintained overnight. The reaction was quenched with saturated aqueous NH_4_Cl (50 mL), and the aqueous layer was extracted with three 50 mL portions of EtOAc. The combined organic extracts were dried (MgSO_4_), concentrated *in vacuo*, and chromatographed (15–25% gradient, EtOAc-hexane) to provide of pure **3** (9.7 g, 78%): ${\left[\alpha \right]}_{D}^{25}$ = +104.4° (c 0.28, MeOH); ^1^H-NMR (500 MHz, CDCl_3_) 0.89 (t, *J* = 6.8, 3H), 1.22 (d, *J* = 6.9, 3H), 1.27–1.40 (m, 4H), 1.40–1.44 (m, 1H), 1.72–1.76 (m, 1H), 2.77 (dd, *J* = 13.3, 9.6, 1H), 3.26 (dd, *J* = 13.3, 2.9, 1H), 3.38–3.73 (m, 1H), 4.15–4.21 (m, 2H), 4.65–4.69 (m, 1H), 7.21–7.34 (m, 5H); ^13^C-NMR (125 MHz, CDCl_3_): 14.0, 17.4, 22.7, 29.5, 33.2, 37.7, 37.9, 55.4, 66.0, 127.3, 128.9, 129.5, 135.4, 153.1, 177.4. ESI-MS: *m/z*: 290 (M+H), 276.

*(S)-2-Methylhexyl 4-methylbenzenesulfonate* (**5**). To a cooled (0 °C) suspension of LiAlH_4_ (606 mg, 16 mmol) in anhydrous THF (20 mL) was added a solution of the imide **3** (1.4 g, 5 mmol) in THF (30 mL) over a 15 min period. After an additional 30 min of stirring, the cold (0 °C) reaction was slowly quenched with water (0.6 mL), then 10% aqueous NaOH (1.2 mL) and water (1.8 mL) to precipitate the aluminum salts, which were then filtered. The filtrate was dried (MgSO_4_), and the solution was concentrated *in vacuo*. Because of the volatility of the product, the crude product was used directly for the next step. To the cooled (−10 °C) solution of the above alcohol **4** in pyridine (20 mL) was added 4-methylbenzene-1-sulfonyl chloride (1.0 g, 5.1 mmol). After the solution was stirred an additional 30 min at −10 °C, the reaction mixture was slowly warmed to 20 °C for an additional 30 min period. The reaction was then quenched with brine (30 mL), and the aqueous layer was extracted with three 30 mL portions of CH_2_Cl_2_. The combined organic extracts were washed with brine, diluted HCl and saturated aqueous CuSO_4_ and dried over MgSO_4_, concentrated *in vacuo*, and chromatographed (10% EtOAc-hexane) to provide pure **5** (0.75 g, 55% for two steps) ${\left[\alpha \right]}_{D}^{25}$ = −2.6° (c 1.67, CH_2_Cl_2_); ^1^H-NMR (500 MHz, CDCl_3_) 0.85 (t, *J* = 7.0, 3H), 0.87 (d, *J* = 6.7, 3H), 1.09–1.13 (m, 2H), 1.20–1.25 (m, 4H), 1.73–1.79 (m, 1H), 2.45 (s, 3H), 3.80 (dd, *J* = 6.5, 6.4, 1H), 3.88 (dd, *J* = 5.6, 5.7, 1H), 7.34 (d, *J* = 8.1, 2H), 7.787(d, *J* = 8.1, 2H). ESI-MS: *m/z*: 270 (M+H), 173, 155, 91.

*9-Bromononan-1-ol* (**7**). A mixture of nonane-1,9-diol **6** (24 g, 0.15 mol), a catalytic amount of iodine (0.5 g, 2 mmol) and 40% HBr (33 mL) in toluene (240 mL) was heated to reflux and the water formed was separated continuously for 30 h. Then the mixture was washed successively with water, aqueous NaOH, HCl, water and brine. The organic phase was concentrated *in vacuo*, and chromatographed (10% EtOAc-hexane) to give 30 g of pure **7** as a colorless oil in 89% yield. ^1^H-NMR (500 MHz, CDCl_3_) 1.31–1.36 (m, 8H), 1.41–1.43 (m, 2H), 1.49 (br s, 1H), 1.53–1.59 (m, 2H), 1.82–1.88 (m, 2H), 3.41 (t, *J* = 6.85, 2H), 3.63(t, *J* = 6.62, 2H). ESI-MS: *m/z*: 223 (M+H), 207.

*2-((9-Bromononyl)oxy)tetrahydro-2H-pyran* (**8**). To a solution of 9-bromononan-1-ol (**7**, 25 g, 0.112 mol) in anhydrous CH_2_Cl_2_ (150 mL) was added 4-methylbenzenesulfonic acid (0.3 g) and freshly distilled 3, 4-dihydro-2*H*-pyran (11.24 mL, 0.123 mol) in CH_2_Cl_2_ (10 mL), at 0 °C. After addition, the mixture was allowed to stir for 5 h at room temperature and monitored by TLC. Then the solution was diluted by addition of another portion of CH_2_Cl_2_ (150 mL) and then the reaction mixture was washed with saturated aqueous NaHCO_3_ and brine, dried over anhydrous MgSO_4_ and then concentrated *in vacuo* and chromatographed to afford 31.6 g (92% yield) of pure **8** as a colorless oil. ^1^H-NMR (500 MHz, CDCl_3_): 1.36 (br s, 8H), 1.36–1.42 (m, 2H), 1.50–1.60 (m, 6H), 1.69–1.71 (m, 1H), 1.80–1.88 (m, 3H), 3.36–3.42 (m, 3H), 3.48–3.51 (m, 1H), 3.36–3.75 (m, 1H), 3.85–3.89 (m, 1H), 4.57 (t, *J* = 3.4, 1H); ^13^C-NMR (125 MHz, CDCl_3_) 19.7, 25.5, 26.2, 28.2, 28.7, 29.4, 29.4, 30.8, 32.8, 34.0, 62.3, 67.6, 98.8. EI-MS: *m/z*: 305 (M-H).

*2-(Dodec-11-en-1-yloxy)tetrahydro-2H-pyran* (**9**). Under a nitrogen atmosphere at 20 °C, Mg turnings (56 mmol, 1.37 g), a catalytic amount of iodine and anhydrous THF (25 mL) were placed in a dry 500 mL three-necked round-bottomed flask equipped with a condenser and a dropping funnel. A solution of compound **8** (13 g, 42 mmol) in THF (150 mL) was added dropwise to the above mixture (approx. 20 min). Then the mixture was allowed to reflux for another 5 h. Another dry 500 mL three-necked round-bottomed flask was charged with allyl bromide (84 mmol), NMP (4 mL) and Li_2_CuCl_4_ (0.3 M in THF, 4 mL). The above Grignard reagent was transferred to this flask through a double-ended needle with stirring. After transfer, the reaction mixture was warmed to 85 °C for 12 h. After cooling, the reaction mixture was quenched by dropwise addition of saturated aqueous NH_4_Cl solution. The THF was recovered on a rotavapor and the residue was extracted with ethyl acetate (100 mL × 3). The combined organic extracts were dried (MgSO_4_), concentrated *in vacuo*, and chromatographed (15–25% gradient, EtOAc-hexane) to provide 7.5 g (66%) of pure **9** as a colorless oil. ^1^H-NMR (500 MHz, CDCl_3_) 1.27–1.30 (m, 10 H), 1.40–1.57 (m, 4H), 1.58–1.94 (m, 8H), 1.95–2.13 (m, 2H), 3.37–3.38 (m, 2H), 3.68–3.73 (m, 2H), 4.57 (t, *J* = 6.8, 1H), 4.91–4.96 (m, 1H), 5.01–5.23 (m, 1H), 5.74–5.99 (m, 1H), ^13^C-NMR (125 MHz, CDCl_3_) some signals were overlapped. 139.2, 114.1, 98.8, 67.6, 62.2, 31.8, 30.8, 29.8, 29.6, 29.5, 29.3, 26.2, 25.5. EI-MS: *m/z*: 267 (M-H), 191.

*Dodec-11-en-1-ol* (**10**). To a solution of compound **9** (7.0 g, 26 mmol) in MeOH (100 mL) was added a catalytic amount of PPTS with stirring. Then the reaction mixture was heated to 50 °C overnight and monitored by TLC. After completion, the methanol was removed on a rotavapor, And the residue was purified by column chromagraphy (15–25% gradient, EtOAc-hexane) to give dodec-11-en-1-ol (4.3 g, 96% yield) as a colorless oil. ^1^H-NMR (500 MHz, CDCl_3_) 1.27–1.30 (m, 14H) 1.52–1.59 (m, 2H), 2.01–2.06 (m, 2H), 2.10 (br s, 1H), 3.62 (t, *J* = 6.6, 2H), 4.92 (dd, *J* = 12.1, 1.6, 1H), 4.98 (dd, *J* = 17.1, 1.6, 1H), 5.75–5.83 (m, 1H); ^13^C-NMR (125 MHz, CDCl_3_) 139.2, 114.2, 62.90, 33.8, 32.7, 31.9, 29.7, 29.5, 29.4, 29.3, 29.1, 28.9. EI-MS: *m/z*: 184.

*12-Bromododec-1-ene* (**11**). A dry 250 mL three-necked round-bottomed flask was charged with PPh_3_ (14.7 g, 56 mmol), dichloromethane (80 mL) and CBr_4_ (22.2 g, 67 mmol) and the mixture was allowed to stir for 20 min. To the above solution was added compound **10** portionwise at −10 °C. After completion, the volatiles were removed on a rotavapor, and the residue was purified by column chromagraphy directly to give 12-bromododec-1-ene (**11**, 90% yield) as a colorless oil. ^1^H-NMR (500 MHz, CDCl_3_) 1.30–1.35 (m, 14H) 1.35–1.45 (m, 2H), 1.83–1.90 (m, 1H), 2.02–2.08 (m, 1H), 3.62 (t, *J* = 6.8, 2H), 4.93–4.96 (m, 1H), 5.00 (ddd, *J* = 21.4, 4.6, 1.6, 1H), 5.75–5.83 (m, 1H); ^13^C-NMR (125 MHz, CDCl_3_) 139.2, 114.1, 33.9, 33.8, 32.7, 31.9, 29.7, 29.5, 29.4, 29.3, 29.1, 28.9. EI-MS: *m/z*: 247 (M-H), 245, 191, 189.

*(S)-14-Methyloctadec-1-ene* (**1**). Under nitrogen atmosphere at 20 °C, Mg turnings (0.2 g, 8.4 mmol), a catalytic amount of iodine, and anhydrous THF (5 mL) were placed in a dry 250 mL three-necked round-bottomed flask equipped with a condenser and a dropping funnel. A solution of compound **11** (1.6 g, 6.5 mmol) in THF (50 mL) was added dropwise to the above mixture (approx. 20 min). Then the mixture was allowed to reflux for another 5 h. Another dry 500 mL three-necked round-bottomed flask was charged with the chiral intermediate **5** (1.8 g, 6.5 mmol), NMP (2 mL) and Li_2_CuCl_4_ (0.3 M in THF, 2 mL). The above Grignard reagent was transferred to this flask through a double-ended needle with stirring. After transfer, the reaction mixture was warmed to 85 °C for 3 days. After cooling, the reaction mixture was quenched by dropwise addition of saturated aqueous NH_4_Cl solution. The THF was recovered on a rotavapor and the residue was extracted with ethyl acetate (100 mL × 3). The combined organic extracts were dried (MgSO_4_), concentrated *in vacuo*, and chromatographed to provide 0.8 g (65%) of pure **1** as a colorless oil. Colorless liquid, ${\left[\alpha \right]}_{D}^{25}$ *=* +1.17° (c 1.82, *n*-hexane). ^1^H-NMR (300 MHz, CDCl_3_): 6.05–5.83 (1H, m), 5.03–4.90 (2H, m), 2.08–2.00 (2H, m), 1.57–1.09 (27H, m), 0.93 (6H, m). ^13^C-NMR (75 MHz, CDCl_3_): 139.3, 114.1, 41.6, 35.2, 34.6, 33.8, 31.9, 29.7, 29.6, 29.5, 29.3, 29.17, 29.0, 22.7, 14.1. EI-MS: *m/z*: 266 (M^+^, 3%), 43 (100%).

## 4. Conclusions

In summary, we have completed an asymmetric total synthesis of (*S*)-14-methyl-1-octadecene, the sex pheromone of the peach leafminer moth, by chiral oxazolidinone auxiliary induced methylation and Li_2_CuCl_4_ catalytic cross coupling reactions. Compared with the reported syntheses, this process has several advantages such as high yield, easy operation and cost-effectiveness. This may be helpful to develop a new generation of affordable pesticides to control peach leafminer moths for Chinese orchardists in the future.

## Supplementary Materials

Supplementary materials can be accessed at: http://www.mdpi.com/1420-3049/18/5/5201/s1.
